# A hole in the T-cell repertoire induced after retroviral infection of immunocompetent adult mice

**DOI:** 10.1186/1742-4690-8-S2-O30

**Published:** 2011-10-03

**Authors:** Masaaki Miyazawa, Shiki Takamura, Sachiyo Tsuji-Kawahara, Eiji Kajiwara, Tomomi Chikaishi, Maiko Kato

**Affiliations:** 1 Department of Immunology, Kinki University School of Medicine, Osaka-Sayama, Osaka 589-8511, Japan

## Background

Acute virus infections resolve following the establishment of host immune responses, and this is also true for infections with most simple retroviruses. Thus, inoculation into neonates and the resultant induction of immune tolerance are required for the development of murine gammaretrovirus-induced leukemia/lymphoma. On the other hand, most complex retroviruses establish persistent infection in immunocompetent adult animals and induce pathologies after a prolonged latent period. Friend virus (FV) composed of two simple gammaretroviruses, replication-competent Friend murine leukemia virus (F-MuLV) and acutely growth-inducing but defective spleen focus-forming virus, is a prominent exception to the above general rules and can induce a rapid development of fatal leukemia when inoculated into immunocompetent adult mice of susceptible strains [1]. Virus antigen-reactive CD8^+^ cells undergo an unusually rapid process of exhaustion in the presence of massively expanding FV-infected cells [2], resulting in profound failure in maintaining FV-specific memory T cells. However, even when acute splenomegaly is resolved in resistant animals, they cannot reject FV-induced leukemia cells and die after tumor cell injection, suggesting that FV-infected mice fail to generate virus antigen-specific naive T cells.

## Methods and results

(C57BL/6 × A)F_1_ (B6AF_1_) mice develop acute splenomegaly, but most recover and survive after FV infection. When injected with FV-induced FBL-3 leukemia cells, uninfected B6AF_1_ mice reject the tumor; however, in FV-infected and recovered B6AF_1_ mice, injected FBL-3 cells continue to grow and cause death within 5 weeks. Syngeneic EL-4 tumor cells irrelevant of FV grow rapidly and cause death, while E.G7 cells expressing an ovalbumin (OVA) epitope are partially rejected, in both uninfected and FV-infected mice. Tetramer staining revealed the expansion of OVA-or influenza NP-reactive CD8^+^ T cells in both FV-infected and -uninfected animals upon E.G7 injection or influenza infection, respectively, while FV antigen-reactive CD8 T cells were not detected in FV-infected mice after FBL-3 injection. Infectious center assays demonstrated the presence of FV-producing cells in the thymus at 1 week after infection, peaking in their numbers at 2 weeks post-infection (pi), and a gradual decrease towards 10 weeks pi. Double negative, double positive, and single positive populations of thymocytes were infected in the thymus. More importantly, thymic dendritic cells and both cortical and medullary epithelial cells expressed F-MuLV envelope protein on the surfaces in infected animals. Transplantation of the thymus from FV-infected mice into thymectomized recipients, but not the transplantation of an uninfected thymus, resulted in a lack of expansion of FV-reactive T cells upon FBL-3 injection, indicating a deletion of the FV antigen-reactive repertoire in the process of T cell development.

## Conclusions

Thus, FV induces virus-specific central tolerance through the infection of thymic antigen presenting cells. This process, along with the early induction of effector T cell exhaustion [2] may contribute to the establishment of persistent infection after virus inoculation into immunocompetent adult mice.

## References

[B1] Miyazawa M Tsuji-Kawahara S Kanari Y Host genetic factors that control immune responses to retrovirus infections Vaccine 2008 26 2981 2996 10.1016/j.vaccine.2008.01.004 18255203

[B2] Takamura S Tsuji-Kawahara S Yagita H Akiba H Sakamoto M Chikaishi T Kato M Miyazawa M Premature terminal exhaustion of Friend virus-specific effector CD8+ T cells by rapid induction of multiple inhibitory receptors J Immunol 2010 184 4696 4707 10.4049/jimmunol.0903478 20351188

